# *In Vitro* and *In Vivo* Investigation of the Efficacy of Arylimidamide DB1831 and Its Mesylated Salt Form - DB1965 - against *Trypanosoma cruzi* Infection

**DOI:** 10.1371/journal.pone.0030356

**Published:** 2012-01-23

**Authors:** Cristiane França da Silva, Denise da Gama Jaen Batista, Gabriel Melo Oliveira, Elen Mello de Souza, Erica Ripoll Hammer, Patricia Bernardino da Silva, Anissa Daliry, Julianna Siciliano Araujo, Constança Britto, Ana Carolina Mondaine Rodrigues, Zongying Liu, Abdelbasset A. Farahat, Arvind Kumar, David W. Boykin, Maria de Nazaré Correia Soeiro

**Affiliations:** 1 Laboratório de Biologia Celular, Instituto Oswaldo Cruz, Fundação Oswaldo Cruz, Rio de Janeiro, RJ, Brazil; 2 Laboratório de Biologia Molecular e Doenças Endêmicas, Instituto Oswaldo Cruz, Fundação Oswaldo Cruz, Rio de Janeiro, RJ, Brazil; 3 Department of Chemistry, Georgia State University, Atlanta, Georgia, United States of America; University of Oklahoma Health Sciences Center, United States of America

## Abstract

Chagas disease is caused by infection with the intracellular protozoan parasite *Trypanosoma cruzi*. At present, nifurtimox and benznidazole, both compounds developed empirically over four decades ago, represent the chemotherapeutic arsenal for treating this highly neglected disease. However, both drugs present variable efficacy depending on the geographical area and the occurrence of natural resistance, and are poorly effective against the later chronic stage. As a part of a search for new therapeutic opportunities to treat chagasic patients, pre-clinical studies were performed to characterize the activity of a novel arylimidamide (AIA - DB1831 (hydrochloride salt) and DB1965 (mesylate salt)) against *T.cruzi*. These AIAs displayed a high trypanocidal effect *in vitro* against both relevant forms in mammalian hosts, exhibiting a high selectivity index and a very high efficacy (IC_50_ value/48 h of 5–40 nM) against intracellular parasites. DB1965 shows high activity *in vivo* in acute experimental models (mouse) of *T.cruzi,* showing a similar effect to benznidazole (Bz) when compared under a scheme of 10 daily consecutive doses with 12.5 mg/kg. Although no parasitological cure was observed after treating with 20 daily consecutive doses, a combined dosage of DB1965 (5 mg/kg) with Bz (50 mg/kg) resulted in parasitaemia clearance and 100% animal survival. In summary, our present data confirmed that aryimidamides represent promising new chemical entities against *T.cruzi* in therapeutic schemes using the AIA alone or in combination with other drugs, like benznidazole.

## Introduction

Chagas disease is a neglected illness caused by the obligatory intracellular protozoan *Trypanosoma cruz*i, extending from Central to South America [1]. This disease has two consecutive clinical phases: acute phase, in which the parasite dissemination can be seen directly on examination of blood. After few weeks, the parasitism burden is controlled by host immune response and the disease moves to the chronic stage. Most of the infected individuals do not present recognizable pathological markers, however, after a long period (about 10–30 years) of clinical latency called the indeterminate form, some of them show disease manifestations, mainly associated with cardiac and/or digestive disturbances [1], [2].

Benzinidazole (Bz) and Nifurtimox (NF), introduced into clinical therapy about 40 years ago, cause many side effects, besides displaying limited efficacy, especially in later chronic phase [2], [3]. Also, several reports have demonstrated that some strains are refractory to treatment [4], [5]. Presently, posaconazole, a new anti-fungal agent that has been effective against *T.cruzi in vitro* and *in vivo* assays, has moved to clinical trials, however, even if effective, its use may be limited due to its high costs [6], [7].

Aromatic amidines (AD) are DNA minor groove binders that recognize enriched AT sequences [8]. In addition to showing high anti-parasitic activity against fungi, amoeba, bacteria and especially protozoan parasites, some of these cationic compounds, such as pentamidine have been used to treat neglected diseases such as African trypanosomiasis and leishmaniasis. Despite having unfavorable characteristics like poor oral solubility and undesirable side effects [9], the broad activity of these compounds has stimulated further screening of new analogs and prodrugs [6]. One class of analogues that have different physiochemical properties are the arylimidamides (AIAs) which have showed high efficacy *in vitro* and *in vivo* against *T.cruzi* [10]–[14]. Studies *in vivo* with the AIA DB766 demonstrated a reduction in the parasite load levels in the blood and cardiac tissue with similar trypanocidal activity as that of Bz in a mouse model of acute *T.cruzi* infection using both Y and Colombian strains [11], [15]. This AIA lead to the recovery of electrocardiographic alterations in addition to reducing hepatic and heart lesions induced by the parasite infection [11]. The excellent activity of DB766 motivated the design and synthesis of novel structurally related compounds including the AIA, DB1831 (hydrochloride salt) and its mesylate salt form (DB1965) for which *in vitro* and *in vivo* studies are reported here with the goal of identifying novel anti-*T. cruzi* candidates for possible future alternative therapies for Chagas disease.

## Materials and Methods

### Compounds

**Figure 1:**
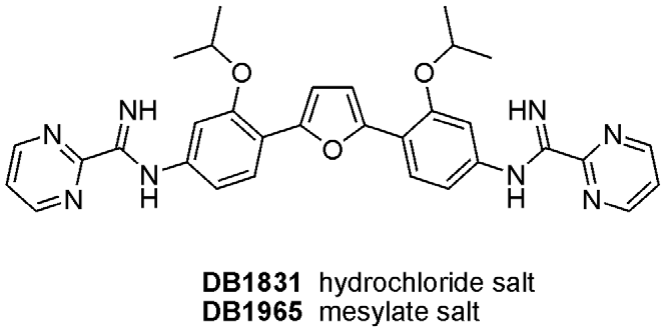
Chemical structure of the compounds.

The synthesis of *DB1831 and DB1965:* (Figure 1) was performed as reported for other analogues ([10], [16] - and will be reported elsewhere). Benznidazole (Bz, Laboratório Farmacêutico do Estado de Pernambuco - LAFEPE, Brazil) was used as reference drug [11]. Stock solutions of the compounds (5 mM) were prepared in dimethyl sulfoxide (DMSO) and fresh final solvent concentration in the assays never exceeded 0.6%, which is not toxic for both parasites and mammalian cells. For *in vivo* studies, a stock solution of DB1965 was first prepared in DMSO and then diluted using distilled and sterile water. The final concentration of DMSO never exceeded 10%, which do not provide detectable mice toxicity [11].

### Cell cultures

For both drug toxicity and infection assays, primary cultures of cardiac cells (CM) were obtained as reported [17]. The cultures were sustained in Dulbecco's modified Eagle's medium (DMEM) supplemented with 10% horse serum, 5% fetal bovine serum (FCS), 2.5 mM CaCl_2_, 1 mM L-glutamine, and 2% chicken embryo extract. Cell cultures were maintained at 37°C in an atmosphere of 5% CO_2_ and air, and assays were run at least three times in duplicates.

### Parasites

Y strain of *Trypanosoma cruzi* (lineage type II) was used throughout the experiments. Bloodstream trypomastigotes (BT) were harvested by heart puncture from *T. cruzi*-infected Swiss mice at the parasitaemia peak day [17]. Intracellular amastigotes lodged within cardiac cell cultures were employed as reported [11].

### In Vitro Cytotoxicity assays

In order to rule out toxic effects of the compounds against mammalian host cells, uninfected cardiac cultures were incubated for 24 and 48 h at 37°C in the presence or absence of each compound diluted in DMEM. The CM morphology and spontaneous contractibility were evaluated by light microscopy. The cell death rates were measured by MTT (3-(4,5-dimethyl-2-thiazolyl)-2,5-diphenyl-2H-tetrazolium bromide) colorimetric assay [18]. The absorbance was measured at a wavelength of 490 nm with a spectrophotometer (VersaMax tunable microplate reader; Molecular Devices), which allows for the determination of LC_50_ (compound concentration that reduces 50% of cellular viability).

### Trypanocidal analysis

BT were incubated at 37°C for 24 h in the presence of increasing non-toxic concentrations of the tested compounds diluted in in RPMI 1640 medium (Roswell Park Memorial Institute- Sigma Aldrich – USA) supplemented with 5% fetal bovine serum. Alternatively, according to protocols already established by our group [11], experiments were also performed with BT for 24 h using serial dilutions of the tested compound at 4°C in the presence or absence of freshly isolated mouse blood (96%). Death rates were determined by light microscopy through direct quantification of the number of live parasites using a Neubauer chamber, and the IC_50_ (drug concentration that reduces 50% of the number of the treated parasites) calculated. For the analysis of the effect against intracellular amastigotes, after 24 h of parasite-host cell interaction (10∶1 parasite∶CM ratio), the infected cultures were washed to remove free parasites and then incubated for another 48 h with increasing but non-toxic doses of the test compounds. CM were maintained at 37°C in an atmosphere of 5% CO_2_ and air and the medium replaced every 24 h. Then, untreated and treated infected CM were fixed and stained with Giemsa solution and the mean number of infected host cells and of parasites per infected cells scored as reported [12]. Only characteristic parasite nuclei and kinetoplasts were counted as surviving parasites since irregular structures could mean parasites undergoing death. The drug activity was estimated by calculating the infection index (II - percentage of infected cells times the average number of intracellular amastigotes per infected host cell) [12]. All assays *in vitro* were run at least twice in duplicates.

### Mice acute toxicity

NOAEL (No Observed Adverse Effect Level) was evaluated using male and female Swiss Webster mice (20–23 g). On day 1, one female and one male mice were treated with DB1965 under two therapeutic schemes: (i) injection via intraperitoneal (ip) every 2 h, with increasing doses, starting at 25 mg/kg up to 400 mg/kg and (ii) administration by ip or per oral (p.o) at doses ranging 25–400 mg/kg [11]. Additionally, male mice (n = 5 for each group) were treated with 20 daily consecutive doses with vehicle (only DMSO and water), 5 mg/kg DB1965 (ip), 50 mg/kg Bz (p.o) and with both combined compounds (5 mg/kg DB1965 ip+50 mg/kg Bz p.o). In all schemes, mice were inspected for toxic and sub-toxic symptoms according to OECD guidelines (Organization for Economic Co-operation and Development). Forty-eight hours after compound injection, the NOAEL values were determined [11].

### Mice infection and treatment schemes

Male Swiss mice were obtained from the Fundação Oswaldo Cruz (FIOCRUZ) animal facilities (Rio de Janeiro, Brazil). Mice were housed at maximum 8 per cage and kept in a conventional room at 20–24°C under a 12/12 h light/dark cycle. The animals were provided with sterilized water and chow ad libitum. Infection was performed by ip injection of 10^4^ bloodstream trypomastigotes. The animals (18–21 g) were divided into the following groups (at least five mice per group): uninfected (non-infected and non-treated); untreated (infected with *T. cruzi* but treated only with vehicle); and treated (infected and treated - ip and p.o - with 12.5 up to 100 mg/kg/day DB1965 or with 100 mg/kg/day benznidazole). For DB1965 treatment, mice received 0.1 mL ip injection or 0.2 mL oral dose, starting at the 5 dpi followed by (i) for 5, (ii) 10 or (iii) 20 consecutive daily doses. For Bz treatment, infected mice received 0.2 mL oral dose (gavage) following the same therapeutic schemes as above described. Thirty days after compound administration, about 1000 µL of blood were collected from the heart of anesthetized mice and then 500, 200 and 250 µL were used for PCR, hemoculture and biochemical analysis, respectively [11].

### Parasitaemia, mortality rates and ponderal curve analysis

Parasitaemia was individually checked by direct microscopic counting of parasites in 5 µL of blood, as described before [19]. At 7, 14, 21 and 28 dpi body weight was evaluated and the data represent the variation between the different mouse groups measured at 30 days post treatment. Mortality checked daily until 30 days post treatment and expressed as percentage of cumulative mortality (%CM) [11].

### Electrocardiography (ECG)

ECG recording and analysis were performed in uninfected, acutely *T. cruzi*-infected mice (after 30 days post treatment) subjected or not to DB1965 and Bz therapy, as previously described [11]. Briefly, mice were placed under stable sedation with diazepan (20 mg/kg, ip), fixed in the supine position, and eight-lead ECG was recorded from 18-gauge needle electrodes subcutaneously implanted in each limb and two electrodes at precordial positions lead II. The electrocardiographic (ECG) tracings were obtained with a standard lead (dipolar lead DII), recording with amplitude set to give 2 mV/1 s. ECG was recorded by using band-pass filtering (Bio Amp - AD Instruments, Hastings, United Kingdom) between 0.1 and 100 Hz. Supplementary amplification and analog-digital conversion was performed with a Powerlab 16S instrument (AD Instruments, Hastings). Digital recordings (16 bit, 4 kHz/channel) were analyzed with the Scope (version v3.6.10) program (AD Instruments). The signal-averaged ECG (SAECG) was calculated by using the mouse SAECG extension (version 1.2) program (AD Instruments) and a template-matching algorithm. ECG parameters were evaluated using the following standard criteria: (i) the heart rate was monitored by beats/minute, and (ii) the variation at P wave and PQ, QRS and QT intervals were measured in milliseconds (ms).

### Blood pressure

Before evaluation of blood pressure, mice were kept in their cages for at least seven days to allow for acclimatization to the laboratory conditions, and a tail sphygmomanometer was fitted for three consecutive readings until stabilization was observed. Blood pressure was individually recorded at 30 days post treatment using an LE 5001 Pressure meter® (PanLab Instruments, Barcelona - Spain), evaluating caudal artery pressure in non-sedated animals. Values of systolic (SP), diastolic (DP) and the mean (MP) pressure were calculated as indicated by the manufacturer [20].

### Biochemical analysis

The levels of alanino Aminotransferase (ALT), urea and creatine kinase (CK) *were determined directly in the blood using the Reflotron system (Roche Diagnostics, F. Hoffmann-La Roche Ltd.; Basel, Switzerland)* as previously reported [11].

### Histopathology analysis

At 30 days post treatment, hearts were removed, cut longitudinally, rinsed in ice-cold PBS, and fixed in Millonig-Rosman solution (10% formaldehyde in phosphate-buffered saline). The tissues were dehydrated and embedded in paraffin. Sections (3 µm) stained by routine hematoxylin-eosin were analyzed by light microscopy. The number of amastigote nests and of inflammatory infiltrates (more than 10 mononuclear cells) was determined in at least 30 fields (total magnification, 40×) for each slide, from at least three mice per group with three sections from each mouse [21].

### Cure assessment

As reported [11], [15], cure criteria were based on three parasitological methods: (i) parasitaemia negativation observed by light microscopy, (ii) Polymerase Chain Reaction (PCR) and (iii) hemoculture assays. Animals presenting negative results for all tests were considered cured.

The DNA extraction and PCR protocols were adapted and standardized for rodent samples as previously reported [11], [15], [22], [23]. Briefly, 500 µL blood was diluted in 1∶3 volume of guanidine solution (guanidine-HCl 6 M/EDTA 0.2 M), and heated for 90 seconds in boiling water in order to cleave the parasite kDNA network [15], [22], [23]. The PCR was performed using the primers: (5′AAATAATGTACGGG(T/G)GAGATGCATGA3′) and (5′GGTTCGATTGGGGTTGGTGTAATATA3′), which amplify a 330 bp sequence from the minicircles kinetoplast DNA (aprox. 120,000 copies/parasite), as previously described [23]. The PCR was carried out using a GeneAmp® PCR Sytem 9700 (Applied Biosystems) as follows: one step at 94°C for 3 min (to activate the Taq platinum DNA polymerase), 2 cycles at 98°C for 1 min and 64°C for 2 min, 38 cycles at 94°C for 1 min and 64°C for 1 min, followed by a final extension at 72°C for 10 min. The amplification products were detected by 1.5% agarose gel electrophoresis following staining with ethidium bromide staining (5 mg/mL). For hemoculture, 200 µL of blood was added to 5 mL LIT medium and incubated at 28°C for 30 days, being weekly examined by light microscopy to detect epimastigote forms [24]. Only negative parasitaemia and hemocultive samples were further screened by PCR analysis [11], [15].

### Statistical analysis

Statistical analysis was performed individually for each assay using a variance (ANOVA) program with the level of significance set at *p*≤0.05. The data are representative of 2–4 experiments run in duplicate.

### Ethics

All procedures were carried out in accordance with the guidelines established by the FIOCRUZ Committee of Ethics for the Use of Animals (CEUA 0028/09).

## Results

DB1831 displayed a dose-dependent trypanocidal activity against bloodstream trypomastigotes, reaching after 24 h/37°C, an IC_50_ value of 20 nM (Table 1). With the goal of possible application in blood bank prophylaxis, BT were assayed at 4°C in the presence or absence of blood constituents. The data at 4°C showed that DB1831 retained a high efficacy (IC_50_ values of 80 and 24 nM with or without mice blood, respectively) as compared to reference drug (IC_50_>250.000 nM) (Table 1). Afterwards, toxicity aspects of DB1831 were studied *in vitro* using cardiomyocytes cultures. Treatment at 37°C for 24 and 48 h resulted in loss of cellular viability only when higher doses were employed, showing 50% lethal concentration of 32 and 15 µM, respectively.

**Table 1:** Trypanocidal effect of Arylimidamides and Benznidazole against *T. cruzi* (Y strain).

Compounds	Bloodstream Trypomastigotes (24 h)				Intracellular Parasites (48 h)	
	4°C		37°C			
	Blood	RPMI				
	IC_50_ (µM)	IC_50_ (µM)	IC_50_ (µM)	SI	IC_50_ (µM)	SI
DB1831	0.08±0.04	0.024±0.004	0.02±0	1600	0.005±0.001	2900
DB1965	0.008±0.00	0.004±0.003	0.031±0.01	342	0.04±0.01	265
Bz	>250	>250	12.94±1.93	>77	2.8±1.96	>360

The activity of the compounds against bloodstream trypomastigotes (BT) and intracellular parasites was evaluated during their incubation at 37°C and at 4°C for 24 h and 48 h.

All assays were run at least two times in duplicate.

IC_50_ values = Compound concentration that reduces the number of parasites by 50%.

SI* = selectivity index corresponds to the ratio LC_50_/IC_50_ – For BT and intracellular parasites calculated on LC_50_ values of 24 and 48 h of incubation at 37°C, respectively.

Bz = Benznidazole.

This AIA was also screened against *T.cruzi*-infected cultures using nontoxic doses (up to 10.6 µM). DB1831 also reduced both the percentage of infected cells and the mean number of parasites per infected cells, revealed by the infection index determination, which exhibited an outstanding IC_50_ = 5 nM after 48 h of treatment (Table 1). Excellent SI values (1600 and 2900 for BT and intracellular parasites) were found (Table 1). However, due to the poor solubility of DB1831, a methanesulfonic acid salt (DB1965) was obtained for further *in vivo* analysis.

Before assaying DB1965 in an experimental model of an acute *T.cruzi* infection, its trypanocidal effect was verified against trypomastigotes and intracellular forms. After 24 h, DB1965 demonstrated high efficacy, showing an IC_50_ = 31 nM at 37°C, with outstanding activity at 4°C in the presence of blood (Table 1). DB1965 was also very effective on intracellular forms with IC_50_ = 40 nM (Table 1). These data confirmed the high activity and selectivity of both AIAs (DB1831 and DB1965) when compared with Bz (Table 1).

Next, two schemes of acute toxicity studies were conducted using DB1965 aiming to determine the NOAEL values. In the first set, when DB1965 was given to female mice by different routes (ip and p.o), considerable toxic side effects like ataxia and tremors (gross pathology also showed hemorrhagic intestinal signs) were found with doses ≥200 mg/Kg administrated by ip, inducing animal death at 400 mg/Kg dose (data not shown). However, oral administration of DB1965 neither lead to mortality nor revealed significant side effects when followed up to 48 h after DB1965 injection (up to 400 mg/kg) (data not shown). In a second scheme of acute toxicity evaluation, female and male mice were injected ip with increasing doses of DB1965. The data confirmed previous results, showing that both animals (female and male) died at the dose of 400 mg/kg, presenting side effect at doses ≥30 mg/kg (Table 2).

**Table 2:** Acute toxicity analysis – Escalating doses using a single mice (starting at 20 mg/kg up to 400 mg/kg DB1965 – ip – using 0.1 mL final volume per mice): Swiss male and female mice (20–23 g).

	20 mg/kg	30 mg/kg	50 mg/kg	100 mg/kg	200 mg/kg	400 mg/kg	NOAEL
Male	No detectable effect	Reversible tremor, abdominal contractions and ruffled fur	Reversible abdominal contractions	Reversible abdominal contractions	Reversible abdominal contractions	Reversible abdominal contractions and death	20 mg/kg
Female	No detectable effect	Reversible tremor, abdominal contractions and ruffled fur	Reversible abdominal contractions	Reversible abdominal contractions	Reversible abdominal contractions	Reversible abdominal contractions and death	20 mg/kg

NOAEL (No observed adverse effect level).

All assays were run at least two times.

Next, efficacy of DB1965 was assayed in Swiss male mice inoculated with 10^4^ bloodstream parasites using three different treatment schemes employing doses that did not cause mortality in the acute toxicity studies. Only those mice that presented positive parasitaemia were used in the following studies.

**Figure 2:**
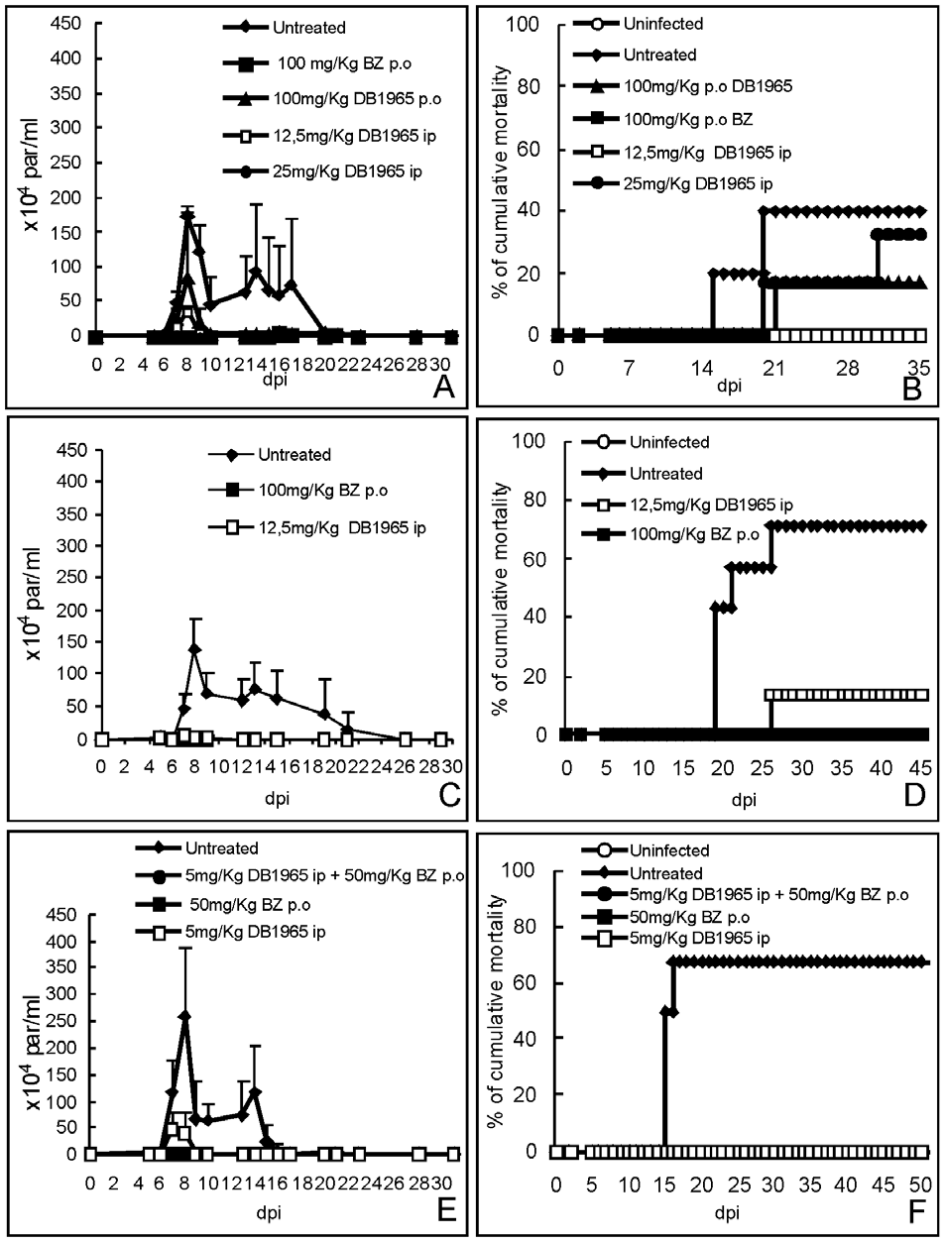
Treatment of *T. cruzi*-infected mice (10^4^ Y strain/mice) with DB1965. The activity of 5 (E–F), 12.5 and 25 (A–D) mg/Kg/day of DB1965 (ip) and 100 mg/kg/day DB1965 via p.o (A–B) is presented. As reference drug, 50 and 100 mg/Kg/day benznidazole (by p.o) was also evaluated using similar therapeutic schemes at the 5–9 dpi (A–B), 5–14 dpi (C–D) and 5–24 dpi (E–F). Parasitaemia curves (A, C and E) and percentage of cumulative mortality (B, D and F) are shown. All assays were run at least two times.

In the first scheme of treatment (Scheme 1), DB1965 was administered at 5 to 9 dpi (5 daily consecutive doses) using 12.5 and 25 mg/kg/day, and 100 mg/kg/day by ip and p.o routes, respectively. As expected for this experimental mouse model of *T.cruzi* acute infection using Y strain, infected and untreated mice (untreated group) presented high parasitaemia levels, peaking at 8 dpi (Fig. 2A). When DB1965 was administrated via ip a reduction of 93 and 99% in parasitaemia levels was observed using 12.5 and 25 mg/kg/day dose, respectively.

On the other hand, gavage administration of DB1965 and Bz resulted in 51 and 100% of decrease in parasitaemia levels, respectively (Fig. 2A).

Biochemical analysis performed at 30 days post treatment showed that only minor differences could be noted between the different mice groups. Regarding ALT measurements a significant increase (*p* = 0.02) was found when infected but untreated animals were compared to uninfected group. However, Bz (*p* = 0.18), 12.5 (*p* = 0.33) and 25 mg/kg (*p* = 0.80) DB1965 did not show any statistically significant difference as compared to uninfected mice (Table 3). When urea levels were analyzed, although no major alteration (*p* = 0.14) was noticed among uninfected and infected but untreated mice, the administration of 12.5 mg/kg DB1965 displayed a statistical difference (*p* = 0.03) as compared to those infected mice that did not receive any treatment (Table 3). On the other hand, neither Bz (*p* = 0.16) and 25 mg/kg DB1965 (*p* = 0.10) showed considerable alteration urea plasma levels as compared to those infected but untreated animals (Table 3).

**Table 3:** Biochemical analysis of *T.cruz*i-infected mice treated or not by DB1965 and Benznidazole.

	Urea (mg/dL)	ALT (U/liter)	CK(U/liter)
Reference Values [references 11], [31]	52±9	22±5	502±507
Uninfected	53±8.4	33±5.6	383±223
Infected and untreated	44±6.7 (*p* = 0.14)	45±5 (*p* = 0.02)	595±622 (*p* = 0.54)
25 mg/kg DB1965 ip	59±11(*p* = 0.1)	43±14 (*p* = 0.8)	208±73 (*p* = 0.34)
12.5 mg/kg DB1965 ip	58±9 (*p* = 0.03)	40±7.6 (*p* = 0.33)	357±61 (*p* = 0.46)
100 mg/kg Bz p.o	56±12 (*p* = 0.16)	35±11 (*p* = 0.18)	296±122 (*p* = 0.45)

All assays were run at least two times and the data represent Mean ±SD.

ALT = alanino Aminotransferase.

CK = creatine kinase.

Bz = Benznidazole.

The analysis of parasitological cure (by hemocultive and PCR) demonstrated that no cure was achieved in both Bz and DB1965 treated mice (Table 4). Also, as the ip dose of 12.5 mg/kg of the AIA achieved 100% of animal survival (Fig. 2B), this later dose was selected for the second round of assays, using 10 daily consecutive doses (at 5 dpi for 10 dpi).

**Table 4:** Cure assessment of DB1965 combined or not with benznidazole (Bz) in murine model of acute *T. cruzi*-infection^1^.

	Experimental groups	Therapy route^1^^,^ 2 ^ and ^ 3	Number of surviving/total number of animals	Assays performed after 30 days post treatment	
				Number of negative hemoculture samples/number of mice	Number of negative blood PCR samples/number of mice
Scheme 1(5 consecutive daily doses)	Untreated	-	3/5	0/3	-
	Bz 100 mg/kg	p.o	4/4	0/4	nd
	DB1965 100 mg/kg	p.o	5/6	0/5	nd
	DB1965 100 mg/kg	ip	3/5	0/3	nd
	DB1965 12.5 mg/kg	ip	6/6	1/6	0/1
Scheme 2(10 consecutive daily doses)	Untreated	-	2/7	0/2	nd
	Bz 100 mg/kg	p.o	5/5	0/5	nd
	DB1965 12.5 mg/kg	ip	7/8	0/7	nd
Scheme 3(20 consecutive daily doses)	Untreated	-	2/6	0/2	nd
	Bz 50 mg/kg	p.o	6/6	0/6	nd
	DB1965 5 mg/kg	ip	6/6	0/6	nd
	DB1965 5 mg/kg+Bz 50 mg/kg	ip+p.o	6/6	1/6	0/1

1 Swiss male mice weight 20 to 24 g inoculated with 10^4^ blood trypomastigotes (Y strain).

Treatment was initiated at 5° dpi followed by different schemes of treatment (up to 20 consecutive daily doses). All assays were run at least twice.

2 Intraperitoneal – ip.

3 per oral – p.o.

Nd = not done.

The data showed that quite similar parasitaemia control was reached using Bz (99.8%) and DB1965 (97%) (Fig. 2C). Although both DB1965 and Bz were effective in protecting against animal mortality, resulting in about 90 and 100% of survival, respectively (Fig. 2D), neither were able to produce parasitological cure of the animals (Table 4). Because some reversible side effects like hyperactivity was noted for the DB1965 group at the end of the treatment (after the 7th day of DB1965 administration), and aiming to find an alternative scheme of therapy using this highly active AIA, a combined treatment of DB1965 (5 mg/kg/day) and Bz (50 mg/kg/day) was next employed, under a scheme of 20 daily consecutive doses.

Our data showed that all treated mice (treated with each compound alone and with Bz+DB1965) presented 100% of survival (Fig. 2F). These treated mice also displayed a suppression of the parasitaemia at the peak day exhibiting 100, 100 and 84% of decrease after Bz, Bz+DB1965 and DB1965 administration (Fig. 2E). The ponderal curve shows that neither DB1965 alone nor in combination with Bz was able to lead to recovery of the mice weight loss induced by the parasite infection (data not shown). The analysis of organ weight revealed that although parasite infection induced an increase in all studied organs (heart, spleen, liver and kidney), only statistically significant differences were observed in liver weight (*p* = 0.022) from infected and untreated mice as compared to uninfected animal groups (data not shown). As compared to untreated mice, all treated groups – Bz (*p* = 0.029), DB1965 (*p* = 0.013) and Bz+DB 1965 (*p* = 0.022) lead to a return of heart weight to pre-infection values (data not shown). Regarding liver, only the combined therapy partially restored (*p* = 0.03) the organ weight increase due to parasite infection (data not shown).

ECG analysis showed a statistically significant bradycardia (*p* = 0.02) in infected and untreated mice group as already reported in this experimental model of acute *T.cruzi* infection [21]. However, none of the therapeutic groups were able to avoid this cardiac electric alteration (data not shown). No statistically significant differences were found in blood pressure analysis among all studied groups (data not shown). Also, among all treatment regimens, only 1 out of 06 surviving mice from the DB1965 treated groups (12.5 mg dose – scheme 1 and DB1965+Bz – scheme 3) displayed negative hemocultive. However, both animals displayed positive PCR, showing no parasitological cure (Table 4).

Histopathology analysis revealed that no major differences could be found in the cardiac tissues among the different experimental infected mice groups (data not shown). Aiming to explore the potential cumulative toxicity after a long-term treatment, different clinical and biochemical parameters were evaluated after 20 daily consecutive doses of each compound. Our data showed that all groups presented 100% survival, and that except for the analysis of body weight (variation between mouse groups measured after the end of the treatment), no major toxic side effects could be observed. We found that mice injected with vehicle alone reached 49±11% of body weight gain, while Bz-treated animals showed 25±11% significant decrease (*p* = 0.006) (data not shown).

## Discussion

AIAs belong to a class of amidine compounds with high trypanocidal activity *in vitro* [14] and *in vivo* [11] and the present study confirmed and extended previous observations of their properties.

The evaluation of both *in vitro* and *in vivo* effects of DB1831/DB1965 against *T.cruzi* infection showed their excellent efficacy against bloodstream trypomastigotes and intracellular amastigotes, with high selectivity indexes, confirming previous data using other AIAs [11], [12], [14]. DB1831 exhibited an outstanding effect against intracellular parasites (IC_50_ = 5 nM), which is about 560-fold higher than Bz. The high activity of DB1831 and DB1965 was maintained when BT were incubated at different temperatures and with blood mice, also confirming the promising activity of AIAs for a blood decontamination protocol [11], [14]. Due to the high selectivity indexes for both parasite forms, DB1965 moved to *in vivo* studies of acute *T. cruzi* experimental infection. Since acute toxicity studies showed side effects at doses ≥30 mg/kg via ip, different protocols were performed using non-toxic doses.

Administration of DB1965 by 5 and 10 daily consecutive doses of 12.5 mg/kg gave similar efficacy as Bz. Also, DB1965 did not induce alterations in CK and ALT plasma levels, as also demonstrated by the use of another AIA, the DB766 [11] as well as with other amidines [20], [21]. The analysis by ECG showed that DB1965 alone or associated to Bz did not revert cardiac electric alterations induced by the parasite infection. However, although DB1965 alone or in combination with Bz did not induce, under the present studied therapy schemes, parasitological cure (evaluated by parasitaemia negativation, hemocultive and PCR assays), this AIA as well as the combined therapy suppressed the parasitemia and provided 100% survival of the infected animals. In fact, unpublished data from our group using the same experimental model of acute *T.cruzi* infection (Swiss male mice infected with 10^4^ bloodstream trypomastigotes of Y strain) showed that no parasitological cure could be reached (hemocultive and PCR analysis performed at the 30 day post treatment under cyclophosphamide administration) after 20 daily consecutive doses of 100 mg/kg Bz, (data not shown).

DB1831 is an analog of DB766, a AIA that presents high efficacy against *in vitro* and *in vivo* experimental models of *T.cruzi* [11] and *Leishmania* [16] infections but showing low activity against *Besnoitia besnoiti in vitro* [25]. Although AIAs also contain amidine groups, they have lower pKa values and thus are more hydrophobic than classical AD since in AIAs an amidine nitrogen atom is bound to an aromatic unit [26]. DB766 (IC_50_ = 60 nM against bloodstream forms) is a modified version of furamidine (DB75) that only displays a moderate anti-*T.cruzi* effect against bloodstream forms (IC_50_ = 16 µM) [27] confirming that small modifications of the chemical structure of these synthetic compounds can lead to a higher selectivity and efficacy. In DB766, the 2 core structure-benzene rings of DB75 were altered through the addition of two iso-propoxy groups, leading to superior effect against intracellular trypanosomatid parasites like *Leishmania* [16], [26] and *T.cruzi* [11], [15]. Similarly, DB1831 and its mesylate form (DB1965) also have high anti-*T.cruzi* activity and selectivity *in vitro* and *in vivo.* The only difference in structure between DB766 and DB1831 is the terminal groups (pyridine and pyrimidine, respectively); which suggests that both pyrimidine and pyridine units in these systems are advantageous for *T. cruzi* activity and merits further investigation. Although treatment with 12.5 mg/kg of DB1965 for 10 days suppressed the parasitaemia and gave 90% protection against mortality, due to the detection of some undesirable sides effects (like hyperactivity), longer periods of therapy (>10 daily consecutive doses) were not performed and a combined treatment of 5 mg/kg DB1965+50 mg/kg Bz (sub-optimal dose) was chosen following a protocol previously established [15]. When comparing the efficacy of DB766 and DB1965 our data demonstrated that this later AIA was not as effective *in vivo* as DB766, especially by p.o route [11]. Since in mouse models, DB766 yields NOAEL values of 400 mg/kg for both p.o and ip routes [11], DB1965 seems to be less well tolerated. As above briefly discussed, the difference in toxicity between DB766 and DB1965, like the difference in efficacy, must be attributed to the difference in terminal groups. Further investigations are required to sort out the effect of this small structural change on both efficacy and toxicity. It is important to note that histopathological and biochemical data gave no major signals of toxicity for DB1965 in the different schemes of treatment employed, using doses up to 100 mg/kg via p.o and 25 mg/kg via ip.

The present report shows the promising *in vitro and in vivo* activity of arylimidamides like DB1965 against *T. cruzi* infection and validates further exploration of AIAs as new candidate for Chagas disease therapy. In fact, although DB1965 did not produce parasitological cure rates, its ability to reduce parasite burden and to yield high protection against mortality highlights the efficacy of these AIAs against *T.cruzi.* These results are encouraging because Chagas disease is commercially an unattractive field for the pharmaceutical industry despite a lack of therapeutic options other than Bz and NF whose short comings are well known [28]–[30].
